# Deep Learning on Multi Sensor Data for Counter UAV Applications—A Systematic Review

**DOI:** 10.3390/s19224837

**Published:** 2019-11-06

**Authors:** Stamatios Samaras, Eleni Diamantidou, Dimitrios Ataloglou, Nikos Sakellariou, Anastasios Vafeiadis, Vasilis Magoulianitis, Antonios Lalas, Anastasios Dimou, Dimitrios Zarpalas, Konstantinos Votis, Petros Daras, Dimitrios Tzovaras

**Affiliations:** 1 Centre for Research and Technology Hellas, Information Technologies Institute, 6th km Charilaou-Thermi, 57001 Thermi, Greece; ediamantidou@iti.gr (E.D.); ataloglou@iti.gr (D.A.); sakellariou@iti.gr (N.S.); anasvaf@iti.gr (A.V.); magoulianitis@iti.gr (V.M.); lalas@iti.gr (A.L.); dimou@iti.gr (A.D.); zarpalas@iti.gr (D.Z.); kvotis@iti.gr (K.V.); daras@iti.gr (P.D.); dimitrios.tzovaras@iti.gr (D.T.); 2 Institute For the Future, University of Nicosia, Makedonitissis 46, 2417 Nicosia, Cyprus

**Keywords:** deep learning, multi-sensor, data fusion, UAVs, security, surveillance

## Abstract

Usage of Unmanned Aerial Vehicles (UAVs) is growing rapidly in a wide range of consumer applications, as they prove to be both autonomous and flexible in a variety of environments and tasks. However, this versatility and ease of use also brings a rapid evolution of threats by malicious actors that can use UAVs for criminal activities, converting them to passive or active threats. The need to protect critical infrastructures and important events from such threats has brought advances in counter UAV (c-UAV) applications. Nowadays, c-UAV applications offer systems that comprise a multi-sensory arsenal often including electro-optical, thermal, acoustic, radar and radio frequency sensors, whose information can be fused to increase the confidence of threat’s identification. Nevertheless, real-time surveillance is a cumbersome process, but it is absolutely essential to detect promptly the occurrence of adverse events or conditions. To that end, many challenging tasks arise such as object detection, classification, multi-object tracking and multi-sensor information fusion. In recent years, researchers have utilized deep learning based methodologies to tackle these tasks for generic objects and made noteworthy progress, yet applying deep learning for UAV detection and classification is considered a novel concept. Therefore, the need to present a complete overview of deep learning technologies applied to c-UAV related tasks on multi-sensor data has emerged. The aim of this paper is to describe deep learning advances on c-UAV related tasks when applied to data originating from many different sensors as well as multi-sensor information fusion. This survey may help in making recommendations and improvements of c-UAV applications for the future.

## 1. Introduction

Unmanned Aerial Vehicles (UAVs) or Systems (UAS) (The terms UAVs, UAS, and drones are equivalently used in this document) are becoming a part of citizens’ everyday life. UAVs have proven to be both autonomous and flexible in a variety of environments and tasks and they bring a continuous market increase in a growing number of useful applications. Recent reports [1,2] confirm the proliferation of UAV production worldwide. Today, UAVs are used from government authorities for tasks such as border security, law enforcement, and wildfire surveillance to commercial related tasks used by civilians such as construction, agriculture, insurance, internet communications, and general cinematography. However, the rapid spread of UAVs is generating serious security issues. In recent years, newspapers and mass media have reported dozens of incidents involving UAVs flying over restricted areas and around critical infrastructures or during public events. On December 2018, a UAV was spotted flying close to Gatwick airport ultimately causing the closure of Britain’s second largest airport for 36 h and disrupting 1000 flights. Reports estimated that this incident alone cost £1.4 m and affected the lives of many passengers [3]. Prisons, airports, sporting venues, public buildings, and other sensitive sites are at serious risk, and correctly understanding the multitude of challenges that UAVs present is central for the effective protection of critical infrastructures and citizens.

**Figure 1:**
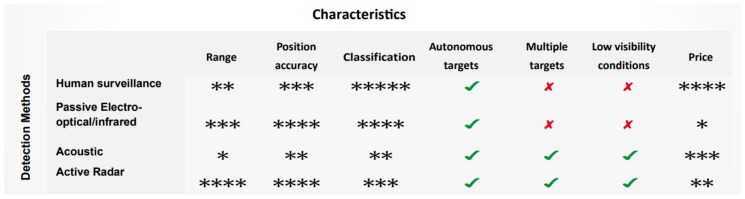
Comparison of key characteristics between individual components of counter-UAV systems.

Recent advances in counter UAV (c-UAV) solutions offer systems [4] that comprise a multi-sensory arsenal in an effort to robustly maintain situational awareness and protect a critical infrastructure or an important event. These applications include multiple integrated sensors for detecting the threat, mainly through radar and/or electro-optical/thermal (EO-IR) sensors and less commonly through acoustic and radio frequency (RF) sensors. Unfortunately, the majority of these systems are commercial applications and elaborating on their specifications would go beyond the purpose of this work. In Figure 1, a general comparison between individual components of such systems in terms of detectable range, localization accuracy, classification capabilities, multi-target extension, and operational conditions with respect to environmental settings and price is presented. Non-automatic systems that include end users who monitor and confirm the classification label of the detected target are usually the best performing in classification capabilities but have generally a high operational cost due to training of personnel and system maintainance. Both electro-optro and thermal cameras offer high classification capabilities with accurate localization and ranging, when multiple sensors are deployed. Electro-optro cameras are generally cheap, though thermal cameras are more expensive, but both are sensitive to environmental settings. On the other hand, acoustic sensors are generally robust to environmental settings, but their limited effective range makes them a less common option. Finally, radar sensors are the most common solution for the detection part due to precise localization and long ranging, combined with decent classification capabilities that operate regardless of environmental settings.

A considerable drawback in multi-sensory c-UAV applications is that the information from the different sensors is not fused to produce a result but instead the alert signals are used independently from each system component to provide multiple early warnings that are later confirmed by a human operator. For example, an early detection coming from the radar sensor is later confirmed by the operator looking at this direction through an optical camera. The system can be fully automatic by leveraging recent advances in data fusion techniques without a considerable trade off in classification capability. Data fusion techniques have gathered significant attention in recent years mainly due to the interest of combining information from different types of sensors for a variety of applications [5]. The target scope of data fusion is to achieve more accurate results than those derived from single sensors, while compensating for their individual weaknesses. On the other hand, artificial intelligence and deep neural networks (DNNs) have become a very attractive methodology for data representation [6]. They are utilized to process a large variety of data originating from many different sources because of their ability to discover high-level and abstract features that typical feature extraction methods can not [7]. Therefore, the utilization of deep learning methods in data fusion aspects can be of significant importance in addressing the critical issue of multi-sensory data aggregation.

A complete c-UAV solution needs to be able to automatically detect the intrusion of potentially multiple UAVs, identify their type and possible payload, and track their movement consistently inside the monitored area. Additionally, multiple information from the available sensors need to be fused to increase the threat’s identification confidence. In summary, multi-object detection and classification as well as multi-object tracking and data fusion are the key tasks at hand. Recent advances with deep learning in several application domains, including generic object detection, salient object detection, face detection, and pedestrian detection are summarized in [8]. Similarly, in [9], an extensive review with the latest advances in tracking algorithms and evaluation of the robustness of trackers in the presence of noise are studied. Finally, Zhu et al. [10] provide a systematic analysis on advances with deep learning on remote sensing applications including data fusion with deep learning. Therefore, many recent scientific publications utilize deep learning based methodologies to tackle these tasks for generic objects showing improvements in performance, yet applying deep learning for UAV detection and classification is considered a novel concept.

**Figure 2:**
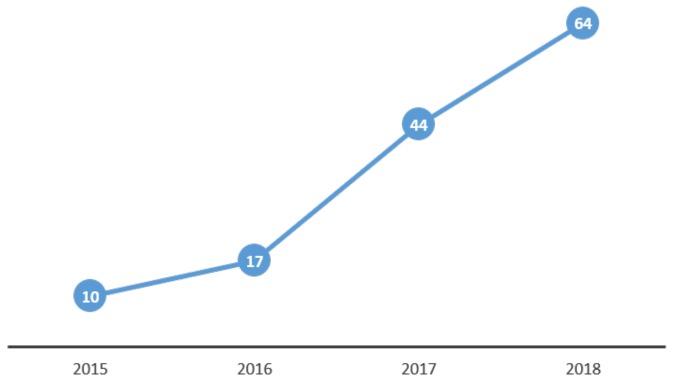
Number of publications with terms UAV or drone detection and/or classification in their title since 2015 based on Google scholar search.

The soaring of UAV production has brought an increase in research publications related to UAV detection and classification over the past few years. Since 2017 more than 100 publications have emerged, whereas, in prior years, fewer than twenty had been published each year. In Figure 2, the number of scientific publications with terms UAV or drone detection and/or classification in their title, excluding patents and citations, since 2015 and up until 2018 based on Google scholar’s search [11], is presented. From January and up until June 2019, another 26 related publications were produced. This steady increase in the number of related publications confirms the valid and increasing motivation of the research community on such task.

In this paper, we focus on the UAV detection and classification related scientific publications which are utilizing radar, electro-optical, thermal and acoustic sensors for the data acquisition and deep learning as the primary tool for data analysis. We specifically select those sensors because they are traditionally included in surveillance systems due to their high performance and can also cover each other’s weaknesses in a potential multi-sensory information fusion scheme. Electro-optical cameras are the most commonly employed sensors for general surveillance. When the target is visible the detection and classification capabilities are the highest. However, occlusions, nighttime and low visibility conditions are the biggest disadvantages. To address some of these issues thermal cameras are often used in combination. Thermal cameras are excellent for nighttime surveillance and depending on the technology they can also “see” through rain, snow, and fog. However, high end thermal cameras are utilized for military applications and the ones found in commercial applications might still face issues with high humidity in the atmosphere or other adverse environmental settings. On the other hand, radar sensors are invariant to environmental settings but may lack in classification capabilities compared to camera sensors. Moreover, high end acoustic sensors are usually robust to environmental settings and provide adequate classification capabilities which make them another reliable choice. Furthermore, we extend our study by presenting recent advances in data fusion methods in order to make recommendations for a potential multi-sensor information fusion based algorithm for increased performance in threat identification. The fundamental aim of multi-sensor learning is to handle and associate information from multiple sensors through a holistic perspective [12]. Dealing with multiple input signals reveals the difficulty of interpreting with a large heterogeneity of data which in most cases results to lack of domain knowledge and data examination [13]. On the other hand, deep learning presents the ability to manage complex and diverse data. Multi-sensor deep learning networks learn features over multiple input streams. Specifically, these networks learn relationships between dissimilar input signals. Moreover, they discover how multi-sensory data can share a representation in a common space [14]. Utilizing deep learning techniques for multi-sensor learning tasks displays major benefits. In particular, multi-sensor learning is capable of understanding in detail real world problems, as well as filling the missing or corrupted sensor data. Consequently, it is obvious that multi-sensor deep learning research constitutes an emerging field, the development of which is critically required to manage the challenging tasks of interpreting, perceiving, and modeling multi-sensor signals.

**Figure 3:**
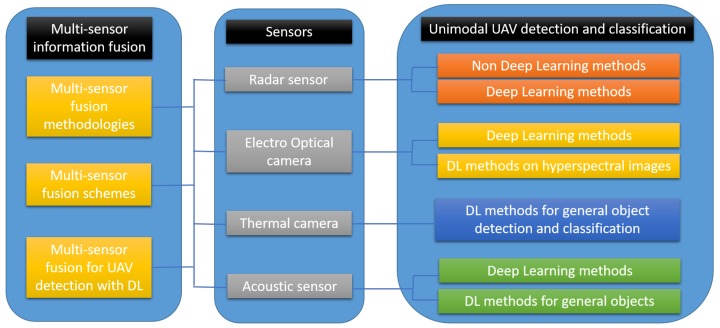
Overall structure of this review.

In Figure 3, we present the overall structure of this review. A section for every selected sensor where the state of the art in uni-modal (analysis on the data originating only on this sensor) UAV detection and classification methods when utilizing deep learning as the main analysis tool is provided. For the case of thermal cameras, we present deep learning based methods for general object detection and classification because, to the best of authors’ knowledge, there is currently no work tackling the UAV detection and classification problem. Finally, a section for multi-sensor information fusion is presented. This section includes a description of the existing multi-sensor fusion methodologies and schemes, and it also reviews scientific publications based on deep learning for the task at hand.

The principal aim of our research is to develop an understanding of the available tools for the task at hand and provide a general road map for the interested reader, as well as make recommendations and improvements on the design of an effective c-UAV system. An essential challenge that remains open and deep learning methods need to solve is the UAV detection and classification problem. In order to contribute to this research, we gather related works that are associated with c-UAV tasks using deep learning methods for following areas: a. Multi sensor fusion, b. Radar sensors, c. Electro-Optical cameras, d. Thermal cameras, and e. Acoustic sensors. The target audience of this systematic review are researchers who focus on UAV detection and classification utilizing deep learning as the primary data analysis tool for each of the aforementioned areas as well as developers from the industrial sector who want to improve their c-UAV applications.

This literature review is defined in the following order. Initially, we focus on the techniques and methods that have been proposed for c-UAV applications and more explicitly in the UAV detection and classification tasks. Nevertheless, several methods that detect, classify and localize UAVs carried crucial importance in recent years. Consequently, we cover both deep learning and traditional non-deep learning based methods for the task at hand. Conventional methods based on handcrafted features and attributes that are associated with the UAV detection and classification task are not as common but are addressed whenever possible to cover the whole picture. Due to the rapid evolution of deep learning, more and more architectures have been proposed, which have the ability to learn high-level and semantic representations. Our taxonomy continues beyond the conventional approaches on UAV sensing, in particular, radar-based techniques or alternative techniques like sound and video-based, as we focus on the advantages and disadvantages of each sensing technologies.

Radar sensors have traditionally been a reliable choice for the detection part, but their classification capabilities are not optimal. Small UAVs are easily mistaken with birds and in most cases, it is hard to distinguish between them. Visual-based techniques utilize high-resolution cameras with the aim to capture UAVs in several backgrounds, but might suffer from occlusions and the distinction between similar shaped objects like birds and the main targets. Thermal vision-based techniques use infrared cameras that take advantage of the heat that electric motors and engines emit. Thermal imaging has gained more and more interest in computer vision since thermal images contain distinctive features about the target object but are generally sensitive to high humidity in the environment. Sound-based techniques make use of arrays of microphones in order to extract the unique acoustic signature of counter UAVs. Typically, flying UAVs provide unique acoustic signatures in a specific frequency range. Acoustic features can be extracted from the time and frequency domain. Sound-based methods can rely on particular audio analysis techniques that are able to extract UAV audio detection from the background noise. Despite all that, training robust deep neural networks require a large amount of training data that in many cases are not feasible. Consequently, our work is motivated by the need to address all the aforementioned challenges. Hence, the focus of our survey is on the deep learning applications on UAV detection, localization and classification tasks.

The rest of this paper is organized as follows: Section 2 investigates research efforts in the context of UAV detection and classification on radar based methods. In Section 3, learning based UAV detection and classification techniques for electro-optical cameras are presented. Section 4 explores applications of deep learning on thermal cameras. In Section 5, learning based UAV detection and classification methods for acoustic data are discussed. Section 6 presents data fusion methods combined with deep learning. In Section 7, a discussion about the impact of the reviewed publications for each topic and a recommendation for an effective c-UAV system is provided. Finally, Section 8 concludes the literature review across the field.

## 2. Radar Sensor

Radar is the traditional sensor for detecting flying vehicles. Compared to other technologies, radar is in principle the only one able to provide long-range detection (from a few kilometers to tens of kilometers, depending on the target radar cross section (RCS) [15]) and almost unaffected performance in adverse light and weather conditions. However, radar sensors designed for detecting standard (manned) aircraft, with relatively large RCS and high velocity, are not suitable for detecting very small and slow moving objects, flying at low altitude such as UAVs [16]. Furthermore, UAVs share key characteristics with birds and reliable classification between the two targets is another key challenge to consider. Therefore, specifically designed radar architectures have been created for this demanding application. The typical detection and classification pipeline is to perform radar signal processing algorithms to detect targets and extract intrinsic features from the processed signal for automatic classification with a machine learning algorithm [17]. Deep learning based pipelines, include processing of the raw data to a more meaningful representation suitable as input to a deep learning network for automatic target detection and classification [18].

In the following subsections, we present recent works in literature tackling the UAV detection and classification task for different radar architectures—initially for traditional machine learning and non-learning based methods and subsequently focusing on recent deep learning based methods. A summary of all the related works discussed throughout this entire section is presented in Table 1. A description of the main radar signal processing methods whenever applied, the feature extraction process, and the employed classifier are provided.

In addition, Table 2 presents the classification results for most of the works included in this review. Unfortunately, a direct comparison for all methods is not possible due to fact that they are evaluated on different datasets, with the exception of [16,24,25] who evaluate under the same data.

### 2.1. Traditional UAV Detection and Classification Methods for Radar Sensors

#### 2.1.1. Micro Doppler Based Methods

The most commonly employed radar signal characteristic for automatic target classification is the micro-Doppler (m-D) signature [40]. The m-D signature has been utilized by many works for automatic target classification such as ground moving target classification [41,42,43], ship detection [44], human gait recognition [45,46], and human activity classification [47,48]. In recent years, it has been an active area of research in the field of c-UAV radar based applications. The intrinsic movements of the targets could describe the rotation of rotor blades of a rotary wing UAV or of a helicopter, the propulsion turbine of a jet, the flapping of the wings of a bird, and can be statistically described by the radar m-D signature [19,20,49]. Publications based on m-D for UAV detection and classification differentiate in the signal processing method to produce the signature, the feature extraction process and the employed classifier.

Among the first who utilized the m-D signature for UAV classification were [19,20]. The authors proposed to produce the m-D signature with spectrogram (Short Time Fourier Transform (STFT)) in [19] and with cepstrogram [50] in [20]. They focused their research on the feature extraction process to produce key characteristics from the radar signal such as rotation rate, blade tip velocity, rotor diameter, and number of rotors to classify between different rotary wing type UAVs. Following a similar approach, Molchanov et al. [16] produced the m-D signature with STFT and extracted eigenpairs from the correlation matrix of the m-D signature as intrinsic features to train three classifiers, a linear and a nonlinear Support Vector Machine (SVM) [51] and a Naive Bayes Classifier (NBC) [52] to classify between ten different rotary UAVs and one class including bird measurements.

De Wit et al. [21] followed a similar signal processing pipeline with [16] before applying Singular Value Decomposition (SVD) to the spectrogram. The authors proposed three main features to allow for quick classification: target velocity, spectrum periodicity, and spectrum width. Similarly, in [22], the authors compared three commonly employed signal representations to produce the m-D signature, namely STFT, cepstrogram and Cadence Velocity Diagram (CVD), followed by an SVD feature extraction step combined with an SVM classifier to classify between real fixed and rotary wing UAV measurements versus artificial bird measurements.

In an attempt to utilize the phase spectrum during the m-D signature extraction, Ren et al. [23] proposed a robust signal representation, namely a 2D regularized complex-log-Fourier transform and an object oriented dimensionality reduction technique, for subspace reliability analysis specifically designed for a binary UAV-classification problem, separating UAVs from birds. Another non common algorithm for m-D signature extraction was proposed in [24]. The authors utilized an empirical-mode decomposition (EMD) [53] based method for automatic multiclass UAV classification. The radar echo signal was decomposed into a set of oscillating waveforms by EMD and eight statistical and geometrical features were extracted from the waveforms. A nonlinear SVM was trained for target class label prediction after feature normalization and fusion. The authors validated their method on the same dataset as [16] outperforming common Fourier based micro Doppler extraction methods. In an extension of [24], Ma et al. [25] studied the usefulness of six types of entropy from a set of intrinsic mode functions extracted from EMD on the radar signal for UAV classification. The authors proposed to fuse the extracted features from the best three types of entropy, obtained with signal down sampling and normalization and then fed as input to a nonlinear SVM. Another work based on both STFT and EMD to extract the m-D signature from a low frequency radar is [26]. The authors studied both the UAV wing type as well as the UAV localization. They handled localization as a classification problem by expanding the number of different classes based on a set number of locations for each UAV type under test. The proposed method combined both EMD and STFT to produce the m-D signature and extracted features with Principal Component Analysis (PCA). The UAV classification and localization was studied under four classifiers, namely a k-Nearest Neighbor, a random forest, a Naive Bayes, and SVM.

Apart from typical radars with one antenna for receiver and transmitter, multi-static radars with more than one antennas are also considered in the literature. In [27], the authors proposed to feed a Naive Bayes and a discriminant analysis classifier [54] with features based on the Doppler and bandwidth centroid of the m-D signatures. The experiments included real measurements of a rotary wing UAV, both loaded and unloaded with a potential payload. In a similar work, Hoffman et al. [28] proposed a novel UAV detection and tracking method on the same multi static radar as [27]. The authors combined m-D features with a Constant False Alarm Rate (CFAR) detector to improve UAV detection results. The detection results are utilized with an extended Kalman filter for tracking. Finally, in [29], the authors utilized two radars operating at different bands to extract and fuse the m-D signatures from both radars in order to classify three different rotary wing type UAVs. The m-D extraction was performed with STFT for both radars and the features were extracted with PCA. The extracted features were fused and fed to an SVM classifier demonstrating gains in performance compared to each radar separately.

#### 2.1.2. Surveillance Radars and Motion Based Methods

A surveillance radar is operating with a rotating antenna to detect and track multiple target. It is designed to constantly seek the space to find new targets [17]. Due to the fact that the radar is constantly seeking the space for new targets, the time used for illuminating the target is usually very small, which does not allow for the m-D extraction [55]. Hence, the classification of the detected targets is performed with features describing the signature of the target, such as RCS, or the motion of the target.

Chen et al. [30] proposed a probabilistic motion model estimation method based on calculating the time-domain variance of the model occurrence probability in order to classify between UAVs and birds with data originating from a surveillance radar. The authors utilized moving direction, velocity, and position of the target information to build their motion estimation models and proposed a smoothing algorithm on top of a Kalman filter tracking to enlarge the gap between the estimations of target model conversion frequency for birds and UAVs. They validated their approach on simulated and real data showing promising results. Torvik et al. [32] considered the UAV versus bird classification problem by using nine polarimetric parameters combined with a nearest neighbor classifier extracted from a signal captured by a surveillance radar. The authors proved that high classification accuracies can be achieved even in the absence of micro-motion features.

Messina and Pinelli [31] study the problem of automatic UAV classification with data originating from a Linear Frequency Modulated Continuous Wave (LFMCW) 2D surveillance radar. The proposed method utilizes a two-step classification process that initially classifies targets between UAV and everything else (e.g., aircraft, birds, humans) and subsequently classifies the recognized UAVs between Rotary wing and Fixed wing type. The proposed classification method is built on [39,56] by creating a set of handcrafted features based on radar signature (RCS, Signal-to-Noise Ratio (SNR), etc), kinematic information (combined with a tracking algorithm for each detected target) and velocity based information. The selected classifier was a SVM that was optimized to avoid overfitting by selecting a subset of the proposed 50 features and by training it under three different schemes. Experimental results show high classification accuracies for both tasks, especially for the first step between UAVs and rest of the world.

### 2.2. Deep Learning Based UAV Detection and Classification Methods for Radar Sensors

Deep learning based methodologies have been the most successful approaches for many tasks in image, video, and audio over the past few years. However, deep learning has yet to take over for tasks concerning radar data. Typical end-to-end deep learning pipelines usually require a large amount of ground truth annotated data that are easily produced and are widely available for common sensors such as cameras and microphones but are scarce for radar sensors. Furthermore, the annotation of radar data can only be performed by an expert in the field. Finally, acquiring raw data from a radar sensor may not be enough since sophisticated radar signal processing is usually required to modify the data representation in order to extract spatiotemporal and intrinsic information which will be meaningful for a deep learning architecture to learn. Despite all these challenges, there has been an increase in works combining radar data and deep learning in recent years. High resolution surveillance radars produce the High Resolution Range Profiles (HRRP) which have been employed by many related works in the literature for large object classification in typical radar architectures ([57,58,59]). Moreover, in [60,61], the authors proposed deep learning based methods for automatic target recognition based on Synthetic Aperture Radar (SAR) images. However, both of these methods require that the targeted objects are fairly large [16], hence are not applicable to UAV detection and classification. The current literature on UAV detection systems that utilize data originating from different radar architectures based on deep learning methods is described here. The main idea is to extract the spectrogram from the radar signal in order to produce the m-D signature of the detected object and utilize the resulting images as an input to a deep learning network [33].

The first work that utilized Convolutional Neural Networks (CNNs) to learn directly from the m-D signature spectrograms and classify UAVs was [33]. The authors employed GoogleNet [62] and trained it with spectrograms from real UAV measurements from a Frequency Modulated Continuous Wave (FMCW) radar. In addition, they proposed a method to improve the shortfalls of m-D signature by merging it with its frequency domain representation, namely the cadence velocity diagram (CVD). They successfully performed tests under two different environments (anechoic chamber and outdoor) with two drones flying under different numbers of operating motors and aspect angles.

Mendis et al. [34] considered a deep learning based UAV classification algorithm developed for a S-band continuous wave radar. They experimented with three different UAV types two rotary and one fixed wing. The spectral correlation function (SCF), which is the Fourier transform of the autocorrelation function, was employed to identify unique modulations caused by the many dynamic components of the target of interest. A deep belief network (DBN) [63] was utilized as the classifier of this work; unlike typical deep neural networks, the layers are interconnected rather than individual units, resulting in a very hierarchical and modular design. The measured data were passed through four SCF pattern reference banks and these were weighted and summed before being fed into the classifier to make a final decision on the target.

Typical radar detection pipelines include Doppler processing [17] and hypothesis testing under the CFAR algorithm. Wang et al. [35] proposed a CNN based target detection algorithm on the Range–Doppler spectrum and compared their method against traditional CFAR detector showing promising results. The target detection problem is generic and does not specify targets to be UAVs, but the same principles can be applied on the UAV detection problem without any constraint. The proposed network architecture was a custom 8-layer CNN trained with different Range–Doppler fixed window segments for targets and clutter under multiple Signal-to-Noise Ratio (SNR) values. The detection problem was handled as a classification task between target and clutter classes where the fixed size window slides over the complete Range–Doppler matrix so that all Range–Doppler cells are checked. The authors validated their method on artificial data simulating that of a continuous wave radar.

A similar work that utilizes the Range Profile and handcrafted features derived from the Range Doppler matrices as input to a DNN for the task of UAV classification is [36]. The authors propose a custom two stream 1D-based CNN architecture that utilizes the Range Profile matrix signature of a detected target and features derived from the Range Doppler matrix such as RCS, SNR, and radial velocity for every detection under test to classify whether it is a UAV or anything else. Experiments on real world radar data collected from a surveillance LFMCW *X*-band radar indicate very promising classification performances.

In an attempt to diversify from the micro motions analysis, Regev et al. [37] developed a Multi Layer Perceptron (MLP) [64] neural network classifier and parameter estimator, which can determine the number of propellers and the blades on a UAV. This is the first work that attempted to learn directly from the received complex valued signal. The network architecture consists of five individual branches, which accept complex in quadrature (IQ), time, frequency, and also absolute data. There are two unique MLP classifiers that first analyze the propeller signatures and then the number of blades; this is then fed into an estimation algorithm. The classification accuracy is very dependent on signal-to-noise ratio (SNR) but is near perfect when applied to synthetic data.

In another body of work, Habermann et al. [38] studied the task of UAV and helicopter classification by utilizing point cloud features from artificial radar measurements. The authors extracted 44 features based on geometrical differences between point clouds. They adopted their feature extraction process based on [65]. The authors trained a neural network with artificial data to tackle two different classification problems, one between seven helicopter types and one between three rotary UAV types.

Finally, Mohajerin et al. [39] proposed a binary classification method to distinguish between UAV and bird tracks with measurements captured under a surveillance radar. The authors adopted a set of twenty features based on movement, velocity, and target RCS extending the works of [56,66] that initially proposed a similar approach to classify aircraft and bird tracks. The handcrafted features are combined with an MLP classifier demonstrating high classification accuracy.

## 3. Optical Sensor

**Figure 4:**
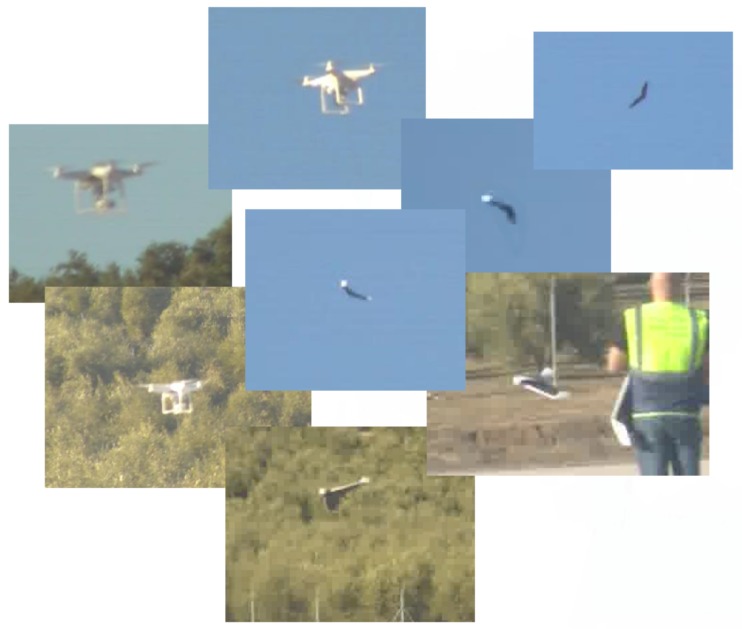
Images with rotary and fixed UAVs at different distances.

With the recent advancements in neural networks and deep learning algorithms, optical data seem to be a really valuable source of information that may provide significant cues to a UAV detection system. Research has evolved around the deep learning paradigm since its great success to classify images on the popular ImageNet dataset [67] at the ImageNet Large Scale Visual Recognition Challenge (ILSVRC) contest in 2012. Most of the works that employ DNNs for determining whether an object is UAV, utilize a generic object detection architecture, with a powerful DNN as a classification model targeted for UAVs. To this end, DNNs are pre-trained beforehand in generic data (like ImageNet) so that can be fine-tuned with UAV data, thus adjusting their parameters for recognizing such objects. Some examples of UAV data captured with a standard visual optical camera are presented in Figure 4.

The work of Saqib et al. [68] examined the Faster-RCNN detection pipeline for the purposes of UAV detection. They carried out many experiments using different baseline models (VGG-16 [69], ZF-net [70], etc.) within the detection pipeline. Their study concluded that the VGG-16 performs best among the other choices for base DNN. In addition, they argued that the existence of birds may challenge the detector performance, by increasing the false positive detections. To address this issue, they proposed that birds cannot be overlooked within training process, but rather they have to be part of the training process as a distinct class, so as to drive the network in learning more fine detailed patterns between UAVs and birds, thereby distinguishing them more efficiently. Moreover, a similar study with more contemporary models as a backbone is carried out from Nalamati et al. [71].

In [72], the authors proposed a VGG based network combined with an RPN to handle small object detection such as birds and UAVs. The network is composed of a convolutional layer, whose output is fed into a classification layer and a bounding box regression layer. The classification layer provides a confidence value about the presence of an object and the bounding box regression layer provides the corresponding coordinates. The authors produced an extensive dataset with images crawled from the web including birds, UAVs, and background photos proving that more diverse data are a winning strategy to further improve the results.

Two recent publications go beyond the classic methods and propose the addition of another sub-module before the detector pipeline, to enhance the representation of the input that is being fed to the detector. In [73], the authors propose the insertion of the U-net before the detector, a network that calculates the motion of successive frames and yields bounding boxes that may contain UAVs with some probability. The final decision on the which boxes belong to UAVs is determined from a Res-net. The other work [74] proposes a sub-module before the detector’s input which performs super-resolution on the initial image, using a deep learning Single-Image-Super-Resolution (SISR) model to enlarge and further improve the initial input representation for the detector. The two models are trained alongside in order to be optimized as a whole model. In doing so, small UAVs that appear too small on the image array—since they usually fly away—can now be detected more easily by the detector, thereby improving the recall performance of the system and thus extend the range detection system’s capabilities.

Another work of Opromolla et al. [75] used traditional computer vision techniques for UAV detection. They employed template matching (TM) using the Normalized Cross-Correlation (NCC) metric to inspect the drone existence. To cope with illumination changes and appearance variations, they applied a morphological filtering at the output of the TM, thus enhancing the detection capabilities for the system, especially for extreme bright or dark UAVs, compared to the surrounded background.

Aker et al. [76] employed a more contemporary object detection pipeline, such as a YOLO (You Only Look Once) detector [77] which enables very fast, yet accurate object detection. Moreover, within the main contributions of the paper is a new artificial dataset they introduced to address the scarcity of annotated public data with drones. To this end, they extracted the background from a number of drones which were found publicly and by keeping only the drone instance, they added it to different natural images with diverse and complex backgrounds. In doing so, they created a sufficient dataset for training a deep learning model with drones at different scales and within various backgrounds.

A different detection framework is proposed from Rosantev et al. [78]. Initially, they split the video sequence in up to 50% overlapping temporal slices. After that, they built spatio-temporal cubes in a sliding window manner for each scale separately. In addition, to yield motion stabilized st-cubes, they performed a motion compensation algorithm to each patch. Finally, the motion compensated patches can be classified as if they comprise any object of interest or not. For classifying the st-cubes, they employed boosted trees and CNNs to infer which method performs better. After extensive experimentation, they concluded that temporal information (motion compensation step) is consequential towards detecting small moving objects like UAVs and CNN perform better in terms of recall–precision metric.

UAV detection with optical cameras that make use of traditional techniques are proposed by Gokcce et al. [79]. They employed traditional features such as Histogram of Gradients (HOG) to describe small UAVs. Moreover, the machine learning detection part utilized a cascaded method of classifier for evaluating at different stages increasingly more complex features, and, if all the stages successfully pass, the object is considered detected. In addition, a Support Vector Regressor (SVR) is trained with distances for each detection, so as to enable distance estimation capabilities at test time.

### Hyperspectral Image Sensors

Hyperspectral image sensors collect information as a set of images across the electromagnetic spectrum. Each image represents a narrow wavelength range of the electromagnetic spectrum, also known as a spectral band. These images are combined to form a three-dimensional (x,y,λ) hyperspectral data cube for processing and analysis, where *x* and *y* represent two spatial dimensions of the scene, and λ represents the spectral dimension (comprising a range of wavelengths) [80]. The goal of hyperspectral imaging is to obtain the spectrum for each pixel in the image of a scene, with the purpose of finding objects, identifying materials, or detecting processes. Hyperspectral imaging applications include the detection of specific terrain features and vegetation [81], mineral, or soil types for resource management [82], the detection of man-made materials in natural backgrounds [83,84], and the detection of vehicles or boats for the purpose of defense and intelligence [85]. These sensors are often mounted on UAVs for airborne object detection applications like agricultural monitoring [86].

To the best of the authors’ knowledge, these sensors have not yet been utilized in a c-UAV application. However, their usage could be a valuable recommendation for such a system. In an urbanized environment, a UAV might fly lower than usual having buildings or ground (e.g., in front of a hill) as its background in order to avoid the effective areas of mainstream sensors like radars. Radars are usually placed on the top of buildings in an urbanized environment to avoid clutter created from other building reflections and also to minimize the exposure of the emitted radiation from the radar antenna to where people live. Hence, a robust c-UAV system needs to be able to address a low flight invasion scenario with its other sensors. This is a challenging case for a traditional RGB (Red Green Blue) or even a thermal camera. Nevertheless, a hyperspectral image sensor could provide appearance cues in any wavelength which are missing in a RGB camera for the prompt detection of the adverse event.

General object detection algorithms on hyperspectral images can be utilized for UAV detection and classification. This would require sufficient data from a hyperspectral image camera capturing UAV flights to fine-tune the existing methods. Prior to the deep learning era, researchers have focused on developing algorithms for target detection on hyperspectral image data using classical detection theory and physics-based signal models. The review in [87] cover developments on such traditional detection algorithms up to 2013. A more recent non-deep learning based method for hyperspectral image reconstruction based on a Markov random field prior, which have labelled a Cluster Sparsity Field (CSF), to model the intrinsic structure of a hyperspectral image probabilistically is presented by Zhang et al. [88]. The authors exploit the spectral correlation and the spatial similarity in a hyperspectral image simultaneously with mining the intra-cluster structures. With the learned prior, the spectral correlation and the spatial similarity of the hyperspectral image are well represented.

With the advancement of deep learning and particularly CNNs, researchers have taken advantage of the more powerful representation capability that CNNs provide and have achieved remarkable results in detecting and classifying objects when using hyperspectral images [81,84,88,89,90]. Paoletti et al. [90] proposed a 5-layered 3D CNN model trained in a specific parallel GPUs (Graphics Processing Units) scheme for training speed optimization, which utilizes all the spatial-spectral information of the hyperspectral image in simultaneous fashion for crops and ground type classification as well as object classification in an urban environment. The authors proved that the joint consideration of spectral information together with spatial information provides better classification results than those reached by traditional neural networks that only include spectral information. Zhou et al. [89] tackled the hyperspectral image classification problem by proposing a discriminative stacked autoencoder comprised in two learning stages which are optimized progressively. The first stage focuses on low-dimensional feature learning and the second stage is used for the joint training of hyperspectral image classifier and feature extractor. The proposed method attempts to learn a low-dimensional feature space, in which the mapped features have small within-class scatter and big between class separation. Extensive experiments in three common datasets and comparisons against other state of the art methods proved the effectiveness of the method.

## 4. Thermal Sensor

**Figure 5:**
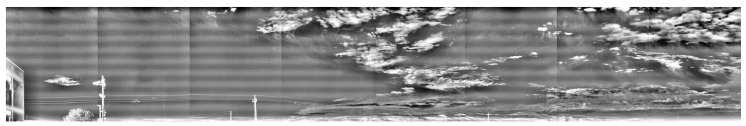
Thermal panoramic image.

Unlike optical sensors, thermal sensors operate in the non-visible electromagnetic spectrum. Thermal cameras are able to capture the infrared radiation emitted by all objects in the form of heat. They are sensitive to the long-infrared range of the electromagnetic spectrum, with a wave length between 9 and 14 μm. The main advantage when using a thermal camera in a security related application is the ability to visualize the surrounding environment regardless of the external lighting or weather conditions and even in total darkness. Furthermore, compared to traditional RGB cameras, thermal cameras offer increased robustness against illumination changes. On the contrary, thermal cameras usually produce lower resolutions images, while being more expensive. Thus, they were initially utilized only in military applications, but recent advances in the technology reduced their cost and allowed their usage in the industry and research sectors. An example of a high resolution thermal panoramic image in depicted in Figure 5.

**Figure 6:**
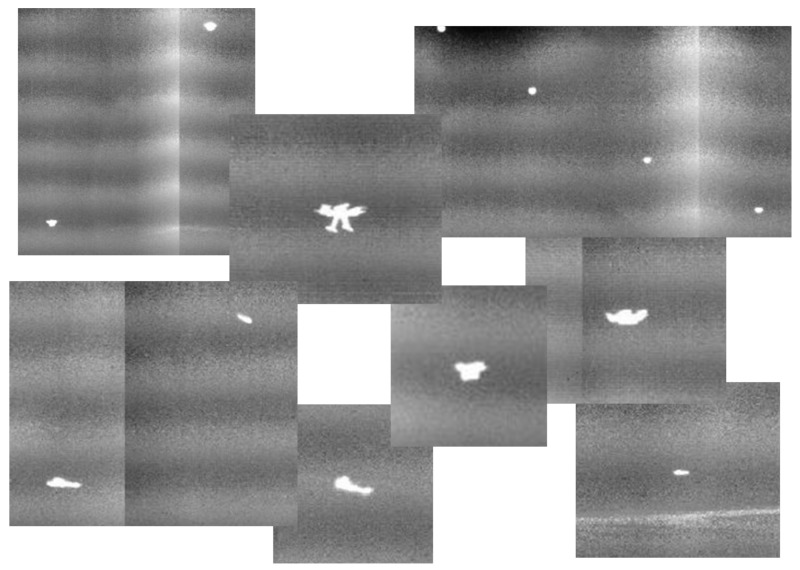
Examples of rotary and fixed UAVs captured by a thermal camera.

In a counter UAV system protecting a secure area, such as a prison facility, thermal cameras are more likely to be positioned either on the ground or on top of other structures (e.g., buildings, surveillance turrets). Some examples of thermal images of captured UAVs are presented to Figure 6. To the best of our knowledge, there is no published work regarding the detection, tracking, or classification of flying UAVs using stationary thermal sensors in such a setting. The most relevant publication to the c-UAV task is [91] where the authors address some of the challenges a thermal camera face against UAVs and propose a localization method via 2D and 3D triangulation for already detected UAV targets when considering images from multiple thermal cameras. Thermal cameras have been successfully employed in other application domains, such as pedestrian tracking or vehicle classification. Furthermore, lightweight thermal sensors have been integrated into UAVs and deployed to several aerial detection or surveillance scenarios. In the rest of this section, we review the recent literature on the utilization of thermal vision in detection, tracking, and classification tasks.

### 4.1. Deep Learning Based Methods Using Thermal Imagery

Most recent research on thermal imagery utilizes deep learning methods, which have proven to be more effective compared to traditional image processing methods. More precisely, recent methodologies usually employ CNNs for solving multiple and diverse tasks. These include the classification of an entire input image or region proposals derived by other methods, the detection and localization of targets within a larger frame or the automatic appearance feature extraction. Depending on the application and the availability of training data, the employed CNNs can be either pre-trained on large generic and multi-purpose datasets, such as ImageNet [92], fine-tuned, or trained from scratch using task-specific data.

#### 4.1.1. Detection

Liu et al. [93] designed and evaluated different pedestrian detection solutions based on the Faster-RCNN [94] architecture and registered multi-spectral (color and thermal) input data. Starting with separate branches for each type of input data, each realized as a VGG-16 [69] base network, they explored feature fusion techniques at a low, middle or high level. Experimental results on the Korea Advanced Institute of Science and Technology (KAIST) Multispectral Pedestrian Detection Benchmark [95] suggested that halfway fusion at a middle level, using concatenation of deep features and the Network-in-Network [96] paradigm for dimensionality reduction led to the best detection performance.

Konig et al. [97] utilized a Region Proposal Network (RPN) for person detection from multi-spectral videos consisting of RGB and thermal channels. Starting with two separate CNNs based on the VGG-16 [69], they fuse the intermediate representations of the optical and thermal inputs halfway in the proposed architecture and generate deep multi-spectral features for the RPN. Deep features corresponding to each region proposal were then extracted from layers both before and after the information fusion, pooled to a fixed size, concatenated and fed to a Boosted Decision Trees (BDT) classifier. The proposed method was evaluated in the KAIST Multispectral Pedestrian Detection Benchmark [95].

Bondi et al. [98] fine-tuned the Faster-RCNN [94] architecture to the task of poacher and animal detection from thermal aerial images. They initialized the VGG-16 [69] base network of Faster-RCNN [94] with a pre-trained weights from ImageNet and subsequently trained poacher and animal specific models using manually annotated videos captured from a UAV.

Cao et al. [99] adapted a generic pedestrian detector to a multi-spectral domain. They utilized complementary data captured by visible light and infrared sensors to both improve the pedestrian detection performance and generate additional training samples without any manual annotation effort. Pedestian detection was achieved using a two-stream region proposal network (TS-RPN), while unsupervised auto-annotation was based on a novel iterative approach to label pedestrian instance pairs from the aligned visible and thermal channels.

Kwasniewska and Ruminski [100] demonstrated how CNNs can be efficiently utilized for face detection from low resolution thermal images, embedded in wearable devices or indoor monitoring solutions for non-intrusive remote diagnostics. Using the concept of transfer learning [101,102], they fine-tuned the Inception v3 [103] model with set of 86k thermal images and modified the final part of the network to enhance it with localization capabilities. This was realized by interpreting the last feature map of Inception as a grid of features and classifying each feature vector independently, thus providing a separate label for each spatial cell in a fixed-sized grid over the input image.

#### 4.1.2. Classification

John et al. [104] utilized a CNN to perform classification of pedestrian candidates. Fuzzy C-means clustering was employed beforehand to segment the input thermal image and localize pedestrian candidates, which were then pruned according to human posture characteristics and the second central moment ellipse. Then, cropped image patches around each candidate were resized to a fixed size and fed to an 8-layer CNN for binary classification, which was trained with a dataset of 16k samples.

Lee et al. [105] used aerial thermal images captured from a flying UAV for early and non-destructive sinkhole detection. Candidate regions were detected by analysing cold spots on the thermal images. Each region was then classified by an ensemble consisting of a light 2-layer CNN for feature extraction followed by a Random Forest for classification, as well as a Boosted Random Forest (BRF) operating with hand-crafted features. Their approach was trained and validated on a limited dataset of eight manually constructed holes with depths ranging from 0.5 m to 2 m, captured from a drone flying at 50 m above them.

Beleznai et al. [106] proposed a multi-modal human detection from aerial views framework, leveraging optical and thermal imaging, as well as stereo depth. The thermal and depth channels were utilized within a shape representation driven clustering scheme for region proposal generation. Afterwards, the proposals were classified as human or other object by two separate classification CNNs, based on the LeNet [107] architecture, each operating either with thermal or optical intensity data.

Ulrich et al. [108] proposed simple two-stream neural networks for the classification of real and mirrored persons, combining images from handheld thermal cameras (often used by fire-fighters) and synchronised micro-Doppler (m-D) radar data. The Viola–Jones [109] method was first used to detect people (either real or mirrored) in the thermal images. Then, an association step between the thermal image detections and the radar targets is performed, using calculated distances from the sensors. Finally, information retrieved from each sensor is first processed and then fused at a feature level within a single joint classifier.

Quero et al. [110] trained shallow two and three-layer classification CNNs intended for the identification of falls in indoor environments. To that end, they composed a dataset of low resolution images captured with a non-invasive thermal vision sensor attached to the ceiling, including cases of standing and fallen inhabitants, as well as images with single and multiple occupancy.

Bastan et al. [111,112] combined a CNN-based detector with a multi-frame classification CNN in an idling car identification framework. Car detection was achieved using the Faster-RCNN [94] architecture, where the VGG base network [69] was fine-tuned twice, first using the Pattern Analysis, Statistical Modelling and Computational Learning (PASCAL) Visual Object Classes (VOC2007) [113] and then with a private dataset of 5670 thermal images of parked cars captured every five seconds in multiple views, with their engine either idling or stopped. Following single-frame car detection, the authors used seven bounding boxes of the same car, uniformly sampled over a 3-minute period, in order to form stacks of cropped thermal images, which were used to model the temporal evolution of the car’s temperature and train a 9-layer binary classification CNN.

#### 4.1.3. Feature Extraction

Liu et al. [114] re-purposed pre-trained CNNs with visible images to the thermal object tracking. They proposed a kernelized correlation filter (KFC) used to construct multiple weak trackers by leveraging features extracted from different convolutional layers of VGG-19 [69] pre-trained on ImageNet, as well as an ensemble method, based on Kullback–Leibler divergence, to fuse the response maps of each weak tracker to a strong estimate of the target’s location. The performance of the proposed framework was evaluated in the Visual Object Tracking Thermal Infrared (VOT-TIR) 2015 and 2106 thermal tracking benchmark datasets [115].

Chen et al. [116] utilized a pre-trained CNN as a feature extractor within a framework purposed for facing direction detection and tracking using a low resolution thermopile array sensor. The first part of a classification CNN, consisting of three convolutional and two max-pooling layers, initially trained for the task of letter recognition, was integrated with an SVM classifier, which was trained using the extracted features. Experimental results showed that CNN-based feature extraction, even when the network is not trained or fine-tuned to the specific domain, outperforms manually defined and extracted features.

Gao et al. [117] proposed a Large Margin Structured Convolutional Operator (LM-SCO) to achieve efficient object tracking based on thermal imaging. Pre-trained CNNs with RGB images were re-purposed to extract deep appearance and motion features of thermal images, which were later fused within the tracking framework. Their method was evaluated in the VOT-TIR 2015 and 2016 thermal tracking benchmarks [115].

#### 4.1.4. Domain Adaptation

Herrman et al. [118] explored different preprocessing techniques, in order to transform input thermal data as close as possible to the RGB domain and thus more effectively reuse pre-trained models on large RGB datasets. Then, following the common practice, they addressed the remaining domain gap by fine-tuning a pre-trained Signle Shot Detector (SSD) 300 [119] detector with limited sets of thermal data. Experimental results on KAIST [95] showed improvements in the task of person detection, derived from both the optimized preprocessing strategy and the adaptation of the CNN-based detector through fine-tuning.

## 5. Acoustic Sensor

**Figure 7:**
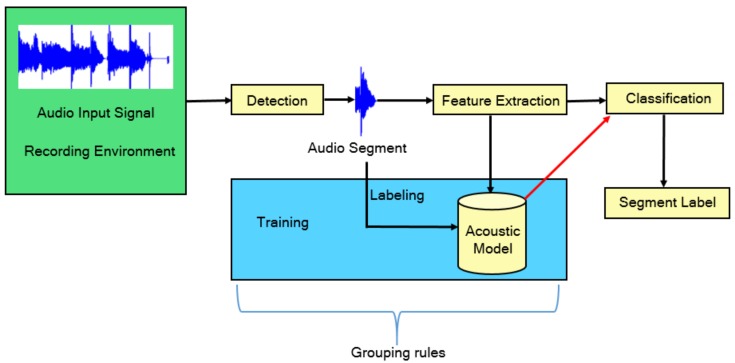
General audio event detection system.

Computational Auditory Scene Recognition (CASR) is a research field that focuses on the context recognition, or the environment recognition, rather than the analysis and interpretation of discrete sound events [120]. Applications of CASR include detection of ambient sounds, intelligent wearable devices, and hearing aids that sense the environment and adjust the mode of operation accordingly. A general audio event detection system (Figure 7) consists of three main modules; *Detection*, *Feature Extraction*, and *Classification*. The *Detection* module refers to capturing the target sound in a real-world noisy recording. The *Feature Extraction* refers to the human engineered features that can be extracted for the features in order to be used as an input to a classifier, or the automatic extracted features from the raw signal when using neural networks. Finally, the *Classification* module assigns the probabilities of the extracted features to the corresponding class.

The ability of deep learning networks to extract unique features from raw data and the high processing speeds of modern Graphic Processing Units (GPUs) lead these networks to receive a lot of attention in a wide range of scientific fields, such as natural language processing, image/video classification and segmentation, reinforcement learning, and audio event detection.

Lee et al. [121] were one of the first ones to introduce unsupervised learning for audio data using convolutional deep belief networks (CDBNs). In particular, they showed that the learned features from the neural networks corresponded to phones/phonemes in speech data. They also showed that these models could be applied to other datasets, such as music genre classification with promising results (comparing to traditional mel-frequency cepstral coefficients (MFCCs) extraction with a classifier). Since then, there were a number of research outcomes in the field of speech recognition [122,123,124,125].

Piczak [126] tested a very simple CNN architecture with environmental audio data and achieved accuracies comparable to state-of-the-art classifiers. Cakir et al. [127] used 1-dimensional (time domain) deep neural networks (DNNs) in polyphonic sound event detection for 61 classes to achieve an accuracy of 63.8%, which was a 19% improvement over a hybrid HMM/Non-negative Matrix Factorization (NMF) method. Lane et al. [128] created a mobile application capable of performing very accurate speaker diarization and emotion recognition using deep learning. Recently, Wilkinson et al. [129] performed unsupervised separation of environmental noise sources adding artificial Gaussian noise to pre-labeled signals and used auto-encoders to cluster. However, background noise in an environmental signal is usually non-Gaussian, making this method to work on specific datasets only.

Over the last few years, many researchers have worked on acoustic scene classification, by recognizing single events in monophonic recordings [130] and multiple concurrent events in polyphonic recordings [131]. Different feature extraction techniques [132], data augmentation [133], use of hybrid classifiers with neural networks [134,135], and very deep neural models [136] have been explored. However, the problem of audio-based event detection remains a hard task. This is because features and classifiers or deep learning approaches that work extremely well for a specific dataset may fail for another.

Regarding the field of audio-based detection of UAVs, researchers have exploited utilizing microphones since the image detection methods contain a few drawbacks. First, the algorithms developed for image detection require high resolution cameras for higher classification accuracy, which results in a trade-off between cost and precision. Secondly, the images captured by these high resolution cameras are significantly affected by the time of the day and the weather. On the other hand, the aforementioned issues can be tackled using low cost microphone arrays with single board computers for the digital signal processing tasks [137]. Other researchers [138,139] proposed drone detection frameworks using audio fingerprints and correlation. The disadvantage of those approaches was that they could not operate in real-time and would work in a very confined dataset. Park et al. [140] proposed a system that used a combination of radar and acoustic sensors and a feed-forward neural network in order to detect and track identifiable rotor-type UAVs. Liu et al. [141] used the MFCCs, commonly used in the field of speech recognition, and an SVM classifier to detect UAVs. Recently, Kim et al. [142] introduced a real-time drone detection and monitoring system, using one microphone. This system used the k-nearest neighbors and plotted image learning algorithms to learn from properties of the Fast Fourier Transform spectra. The authors extended their work [143] and increased the classification accuracy of their proposed system from 83% to 86%, using an artificial neural network. They created a background noise class to separate the drone sounds using the UrbanSound8K dataset [144]. Jeon et al. [145] presented a binary classification model that used audio data to detect the presence of a drone. A Gaussian Mixture Model, a Recurrent Neural Network, and a CNN were compared using the F-Score as a performance metric. This work also showed that the classification performance increased when using data augmentation methods. In particular, they synthesized raw drone sound with diverse background sounds to increase their training data.

There is a strong need for the collection of a real-world UAV audio dataset that could serve as a benchmark for researchers to develop their algorithms. Two-dimensional computer vision neural networks (e.g., DenseNet) should be tested using raw short-time Fourier Transform spectrograms or mel-spectrograms, in order to have a robust UAV audio-based detection system.

## 6. Multi Sensor Fusion

Data fusion from multiple sensors aims to combine data from different modalities to generate inferences that would not be possible from a single sensor alone [146]. It has various applications in the fields of target recognition and tracking, traffic control, UAV detection, remote sensing, road obstacle detection, atmospheric pollution sensing, monitoring of complex machinery, robotics, biometric applications, and smart buildings. The wide variety of information resources in the real world enables multi-sensor data fusion to discover relationships between different sensor type data, learn from them and recognize patterns. The most interesting challenge in data fusion is to achieve a joint representation of multi-sensory data. In recent years, artificial intelligence and deep neural networks have become very attractive in representation of multi-sensory data [147]. In general terms, multimodal learning is cumbersome since the data come in different representations. As a consequence of the multimodal learning challenges, there are some possible solutions such as combining separate learning models for single modalities at a higher and abstract level.

### 6.1. Multi-Sensor Fusion Methodologies

Ngiam et al. [148] described the general scope of multimodal learning. Moreover, they presented how data can share the same representations from different modalities. Specifically, they used a cross modality feature learning method using Restricted Deep Boltzmann Machines [149]. This research has been evaluated in CUAVE [150] and AVLetters [151] datasets on classification purposes combining audio and visual data.

Baltrušaitis et al. [147] presented various multimodal machine learning approaches. The findings of such studies helped to understand the different ways that multi-sensory information, such as image, video, and audio, can be handled. This research analyzed the various challenges that multi-sensor data fusion deals with. The authors illustrated five technical challenges, which are representation, translation, alignment, fusion, and co-learning. Specifically, representation is a learning method that combines unimodal signals into a common representation space. Moreover, translation is defined as the process of changing the form of data from one modality to another. Alignment is the operation where direct relations between elements from various modalities can be identified. Fusion describes a concept that integrates information from multiple sources for the purpose of a performance metric. Finally, co-learning is a technique where knowledge is transferred between modalities.

Liu et al. [14] introduced learning techniques for multisensory information fusion and modalities combination. In particular, they proposed a deep neural network architecture that multiplicatively combines multi-sensory data. The proposed method has been validated in three domains: image recognition using CIFAR dataset [152], physical process classification using HIGGS dataset [153], and user profiling using Gender dataset [154].

One of the most interesting works in data fusion was presented by Dong et al. [155]. This research highlighted image fusion methods with emphasis on remote sensing field. Specifically, the authors analyzed some standard image fusion algorithms, such as Principal Component Analysis (PCA) [156] and IHS [157], whereas they inspected wavelet-based artificial neural networks based methods.

Khaleghi et al. [12] provided a survey about multi-sensor data fusion. This work studied the advantages and the challenges of multi-sensor data fusion, along with the state-of-the-art algorithms. Furthermore, it presented extended methodologies for fusion of defective or corrupted data.

Hu et al. [13] performed a study about a dense multimodal fusion. The scope of that work was to densely integrate representations of different modalities. The authors studied the benefits of joint representations and learned the dependence among hierarchical correlations. The aim of that paper was to employ Dense Multimodal Fusion in the purpose of achieving faster model convergence, lower training loss, and greater performance than common mutlimodal fusion methods.

### 6.2. Multi-Sensor Data Fusion Schemes

**Figure 8:**
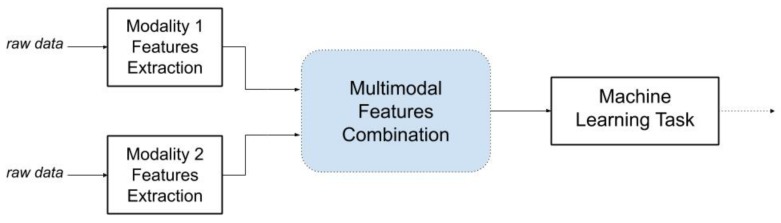
General scheme for early fusion. The outputs of unimodal analyses are fused before a concept is learned.

**Figure 9:**
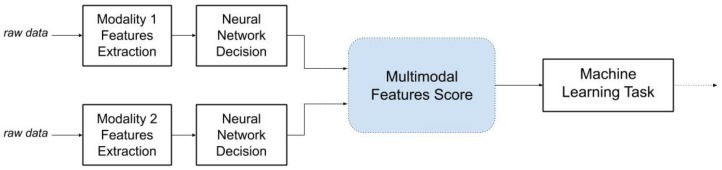
General scheme for late fusion. The outputs of unimodal analyses are used to learn separate scores for a concept. After the fusion procedure, a final score is learned for the concept.

Data fusion technology can be divided into two main categories of fusion methods, namely **Early Fusion** and **Late Fusion**. The choice of the fusion technique can be determined by the requirements of the problem and the sensor types. Early and late fusion differ in the way they integrate the results from feature extraction on the various modalities. Early fusion (Figure 8) yields a multimodal feature representation, considering the features are fused from the start. In particular, early fusion combines several features such as edges, and textures into a feature map. On the contrary, late fusion (Figure 9) focuses on the individual strength of different modalities. Unimodal concept detections are fused into a multimodal semantic representation rather than a feature representation.

Snoek et al. [158] introduced a multimodal video semantic analysis. The authors performed two categories of early fusion and late fusion, where the modalities are fused in a feature space and in a semantic space accordingly. Both methods were validated within the TRECVID video retrieval benchmark [159].

Ye et al. [160] proposed a late fusion based rank minimization. Specifically, the work aimed to predict confidence fusion of multiple models. The main assumption of that work was that relative score relations are persistent between component models. The confidence scores are represented in the form of vectors. The proposed work has been evaluated by utilizing the Oxford Flower 17 dataset [161].

### 6.3. Multi-Sensor UAV Detection

In recent years, the number of unmanned aerial vehicles (UAVs) has been greatly increased. This growth is driven from the vastly expanded number of applications that a UAV can be used for. In this context, there is emerging research regarding object detection methods. However, UAV detection requires the development of more robust systems in order to safely perform identification. The major challenge in UAV detection is mainly the small size to be detected. In addition, there is a possibility that a UAV may not have an authorized and appropriate flight plan.

Bombini et al. [162] proposed a methodology of radar and vision fusion, in order to improve the vehicle detection system’s reliability. The authors perform a radar-vision fusion that is based on a conversion of radar objects into an image reference system. The aforementioned method has been tested in urban environments achieving great results.

Jovanoska et al. [163] presented a research about UAV detection and multi-sensor data fusion based on bearing-only as well as radar sensors for tracking and localization capabilities. A centralized data fusion system was analyzed that was based on the multi-hypothesis tracker [164]. The authors analyzed the behavior of each sensor and studied the advantages of using multi-sensor systems for UAV detection. Its objective was the improvement of the localization of the detected targets by fusing results from disparate sensor systems. The system consisted of radar and bearing only sensors (cameras, radio frequency sensors). Its sensor data fusion scheme was comprised of three main functionalities: data association, target detection, and target localization. It first identified which detections from different sensors belong to the same target and which of them are false alarms. The targets were recognized and the individual sensor’s contribution to the detection was measured. Finally, a fusion algorithm was used for the estimation of the target location.

Hengy et al. [165] proposed a sensor fusion scheme that aimed at detecting, localizing, and classifying incoming UAVs by utilizing optical sensors, acoustic arrays, and radar technologies. The system achieves localization accuracy with mean azimuth and elevation estimation error equal to 1.5 and −2.5 degrees, respectively. Localization from acoustic arrays is achieved using the MUltiple SIgnal Classification MUSIC algorithm and the systems detection capability is enhanced by radar technologies to minimize false alarm rate. Finally, the authors proposed a method that combines images from short-wave infrared (SWIR) and visible sensors in order to achieve easier and faster detection of the UAV in the presence of clutter, smoke, or dense background on the vision sensors.

In [166], Laurenzis et al. investigated the detection and tracking of multiple UAVs in an urban environment. The proposed system is comprised from a distributed sensor network with static and mobile nodes that included passive/active optical imaging, acoustic antennas, LiDAR, and radar sensors. They proposed a fusion method of acoustic information, which was realized by triangulation of the lines of bearing of the acoustic antennas. The method detects a drone and localizes it with a mean error of about 6 meters when the localization results are compared to ground truth data.

### 6.4. UAV Detection Using Multi-Sensor Artificial Intelligence Enabled Methods

Park et al. proposed a system that combines radar and audio sensors [140] for detection of small unmanned aerial vehicles. The system uses ‘Cantenna’, which is a modified version of a handmade radar [167] to detect moving objects in a target area and an acoustic sensor array that determines whether the object detected from the radar is a UAV or not. The system also used a pre-trained deep learning algorithm consisted of three MLP classifiers that vote whenever they receive any acoustic data about the existence or not of a UAV. The system was tested on both recorded and field data and correctly detected all cases where a UAV was present with no false negatives and only few false positives. The estimated costs of each microphone assembly and the radar are quite small, which makes this approach a very cheap solution. However, the system’s detection range is about 50 m, which is limited compared to other UAV detection systems.

A larger installation of a multi-sensor fusion system was described in [168], where the authors proposed an airborne threat detection system combined from low cost, low power netted sensors that included a simple radar, infrared, and visible camera as well as an acoustic microphone array. The system was able to identify and track a potential airborne threat by employing a Kalman filter for associating the multiple sensor data in order to be fed to a nearest neighbor classifier for obtaining the final results. The system was able to accurately track aerial vehicles up to 800 m range, providing also a high modular and adaptive technology setup.

A solution [141] that used both a modular camera array and audio assistance presented results of high detection precision. The system was tested against a dataset of multiple drones flying under various conditions at maximum flight altitudes of 100 m and maximum horizontal distance of 200 m. The system was consisted of 30 cameras, eight workstation nodes, three microphones, and some network devices. An SVM classifier was trained to detect a drone in the image while another SVM was trained to detect the noise produced by the drones.

At [169], a general information fusion framework is proposed, in order to join information from different sources. The major key of this work is to implement a robust algorithm that merges extracted features from deep neural networks. Explicitly, the aim of this work is to efficiently perform a neural network-based algorithm for the UAV detection task.

## 7. Discussion and Recommendations

In this literature review, various deep learning based methods for UAV detection and classification using data from radar sensors, electro-optical cameras, thermal cameras, and acoustic sensors have been thoroughly reviewed. A review on multi-sensor information fusion analysis with deep learning for the same sensors and the same task is also considered. In the following sections, we focus on the impact of the described work from each topic, introduce a comparative analysis with potential limitations and drawbacks, and identify the key objectives for the described methods. Finally, we recommend a c-UAV system, which we believe to be effective against the challenges posed by the misuse of UAVs.

### 7.1. Impact of Reported Studies

#### 7.1.1. Radar Sensor

UAV detection with radar sensors is mainly achieved with the classic radar signal processing for target detection using Doppler processing and hypothesis testing under the CFAR algorithm as it is described in [17]. Alternatively, there is a promising deep learning based method from [35] for general target detection, but the experiments are on artificial data so it is not definitive if this method is applicable in a real world application.

On the other hand, the UAV classification task using radar data is a much more active field of research, and most practices have largely been successful through the transfer of established techniques (deep learning or machine learning based) migrated from other automatic target recognition problems. There are two directions on the UAV classification problem with radar data: methods that utilize the micro-Doppler (m-D) signature and methods that rely on different sources of information such as kinematic data or features derived from the Range Doppler and Range Profiles matrices. Both directions have their merits and flaws that are summarized below. The key objectives that the proposed method should answer to are to minimize false alarm rate and detect the target at all times regardless of the way it moves.

The most commonly employed radar signal characteristic for UAV classification is the m-D signature [16,19,20,21,22,23,24,25,26,28,29,33]. The main contributing factors for the m-D signature are the number of fast rotating blades, the wide range of angles incident to the radar, especially during manoeuvres, the Pulse Repetition Frequency (PRF) of the radar, and the time which the radar is illuminating the target. These factors are important to consider when designing a radar based c-UAV system. The volatile nature of the target can make the reliable extraction of the m-D signature not a trivial task. Most of the discussed work has been undertaken within ideal scenarios and usually at close range (250 m furthest [19], 30 m furthest [16,24,25]), certainly having an effect on classification performance. A further complication to the research is that not all of the aforementioned works evaluate on original radar data with many conclusions being drawn on artificially created datasets trying to emulate a UAV signature [38,39]. However, this is because radar sensors specialized for small target detection, which usually operate at an *X*-band are not easily accessible to a university or a research centre, and currently there is no publicly available dataset on UAV detection and classification with radar data for researchers to develop and evaluate their methods.

To address some of these issues, techniques that employ different sources of information such as motion and RCS related features derived from the Range Doppler and Range Profiles matrices are of great interest [31,32,36,39]. Such data are produced by surveillance radars that provide 360${}^{\circ }$ coverage of the protected area with a rotating antenna. Surveillance radar can not produce the m-D signature because they do not illuminate the target long enough, hence they do not rely on micro motions of the target for target classification. Trajectory classification is another successful approach to differentiate between targets in such cases [30]. However, this area of research is rather underdeveloped compared to m-D based approaches which could encourage further work to be made. In particular, deep learning based methods for trajectory classification have been successfully studied for general motion models [170,171] and such techniques could transfer to UAV trajectory classification making the first step towards that research field. As radar systems become increasingly adaptive, it is safe to assume that they will be able to fuse more information to enhance their classification capabilities, whilst also exploiting the latest developments from the deep learning community.

#### 7.1.2. Optical Sensor

Standard optical cameras are easily accessible to everyone and general object detection methods for images and videos are well established since this is a very mature research field. The adaptation of existing methods in the UAV detection and classification problem has already emerged with many c-UAV related publications referring to common deep learning based object detection architectures such as Faster RCNN [68,74], SSD [71], and YOLO [76]. There are also publicly available datasets for UAV detection and classification when using a standard RGB cameras [172,173,174], which is another proof to how accessible and mainstream optical sensors are. Finally, UAV detection and classification challenges when using an optical camera are organized at workshops [175,176,177] in major conferences such as the International Conference on Advanced Video and Signal-based Surveillance (AVSS) and the International Conference on Computer Vision Systems (ICVS). Thus, it is clear that research on optical sensor data are in a thriving state and the existing methods are expected to continue evolving.

Most successful approaches focus on deep learning since traditional methods from computer vision with handcrafted features [75,79] can not achieve comparable performances. The key objectives that the proposed method should answer to are accuracy and speed. The target is to minimize false alarm rate and run the method at real time. When comparing the Faster RCNN [68], SSD [71], and YOLO [76] architectures, YOLO is the fastest and Faster RCNN is the most accurate. SSD can be a good choice only when the objects are large, which is rarely the case for a c-UAV application. The work of [71] compared Faster RCNN with SSD for the UAV detection and classification problem, both with Inceptionv2 as their backbone network architecture, and concluded that Faster RCNN has a better accuracy, but SSD is faster. However, the research does not stop on migrating existing general object detection methods on c-UAV data. Researchers are actively trying to improve these methods by looking at the challenges that UAVs present. The small shape and unique manoeuvres are being taken into consideration by utilizing temporal information across multiple consecutive frames [73] or by adding super resolution techniques [74] in an effort to avoid false positive detections and detect very small objects.

In summary, general object detection architectures based on deep learning seem to work for the UAV detection and classification problem but do not achieve the optimal results which has made researchers to explore different information cues (e.g., temporal information, super resolution) in order to improve existing methods. A very interesting future work would be a combination of existing design elements, such as deep learning based detector combined with temporal information and super resolution.

#### 7.1.3. Thermal Sensor

Even though thermal cameras are very common in c-UAV systems, the related scientific work in UAV detection and classification is nearly non-existent. This is attributed to the fact that the widely available thermal sensors in commerce usually produce low resolution images which present a major challenge in detecting small objects such as UAVs. At the same time, higher resolution thermal cameras are quite expensive and they are not easily accessible to the research community. Consequently, the creation of a dataset for UAV detection and classification based on thermal images without an increased budget might be out of reach for many universities and research centers.

Nevertheless, deep learning based object detection algorithms developed for normal RGB images have been successfully applied on thermal images for other problems such as pedestrian detection [93,97,104] or idling cars detection [111,112]. Of course, the main characteristics of humans and cars are vastly different from UAVs, but, if the target is visible, then these methods should also work for UAVs. The key objectives that the proposed method should answer to are the same as that of electro-optical cameras, accuracy, and speed. The prevalent method in literature for object detection and classification with thermal images is the Faster RCNN architecture combined with VGG as its base network [93,98]. While this approach is designed to detect and locate the target within a larger frame, it does not utilize the information that multiple consecutive frames contain. Hence, the addition of the detection and classification across time might fill in some of the gaps that low resolution images and small shape of targets present.

#### 7.1.4. Acoustic Sensor

Despite being low-cost type of sensors, acoustic sensors are prone to noise. The main limitations of the aforementioned research are related with the number of microphones, used for the data collection, and the distance of the acoustic sensor from the UAV. Additionally, there is a strong need for a public dataset that includes sound signals from UAVs, in order to develop robust algorithms for the particular task of drone detection.

Regarding the number of microphones needed for UAV detection and the distance of the acoustic sensor from the UAV, past research [145] has shown that it is not possible to detect a drone’s sounds at distances greater than 150 m. Furthermore, studies regarding multi-channel audio [141] have shown that it is possible to significantly increase the performance of a detection framework, when using state-of-the-art beam-forming algorithms and fusion with other sensors (e.g., cameras). Regarding future research, it would be possible to transfer knowledge from the domain of speech recognition [178], using far-field signal processing techniques.

Finally, creating a public database with drone sounds can be an expensive task. It requires many data capturing sessions of drones flying at various distances from the sensor, at various sampling rates and bit depths. Labeling such a dataset can be prone to human error, leading a machine algorithm to learn from the wrong labels. Towards this end, unsupervised learning algorithms (generative and discriminative) [135] have recently received great research interest, especially in the field of environmental sound detection. Therefore, these algorithms could be adapted for the problem of drone detection, increasing the recognition accuracy, since they can be used for data augmentation (generative) and for clustering (discriminative).

#### 7.1.5. Multi-Sensor Information Fusion

The real-world experience involves information from several sources. Multi-sensor deep learning provides a wide variety of application, for instance, audio-image translation, image fusion for object detection. The concepts of Deep Learning can be easily related to the fusion of multimodal information on the grounds that Deep Neural Networks have the power to learn a high-level representation of data [147]. This fact results in achieving robust and in most cases the most optimal characterization of the raw information. There are remarkable achievements of multimodal deep learning methods on solving detection tasks. Although many deep learning techniques have demonstrated considerable attention in vehicle detection tasks, the studies to detect explicitly UAVs have not yet taken advantage of them.

An overall UAV detection system should be able to identify targets in several conditions such as a possible presence of sensor noise, different flight range, elevation, or azimuth. Single sensor cases can not ensure reliable detections. The single sensor observations may be noisy or incomplete. There can be no doubt that multi-sensor information fusion techniques are applicable to UAV detection tasks. UAVs can fly in different conditions such as urban or remote environments. In real UAV flight scenarios, it is necessary to exploit a multi-sensor fusion sensing system in order to adjust UAV detections in changing environments and achieve the greatest feasible localization of the targets [163]. Multi-sensor information fusion can be implemented either constructing complicated architectures that process raw multimodal data [140,141,168] or design novel frameworks that handle high-level representations of multimodal data [169].

Meanwhile, there are some primary issues that are in great need of resolution. First of all, a proper and efficient way to join information coming from several sources should be found. In many circumstances, this information contains lots of noise. Moreover, multi-sensor information is recorded using different sensor configuration which means that the raw information will be diverse in its representation. In addition, diverse representation probably leads to different predictive power.

A variety of multi-sensor methodologies applicable to vehicle detection tasks have been proposed. Each methodology has its features that have an effect on relevance in the UAV detection problem. Multi-sensor information fusion can without difficulties complement more common methods, such as optical and thermal cameras, acoustic arrays, and radar sensing systems in order to tackle counter UAV detection challenges. Multi-sensor data fusion is capable of providing considerable advantages over single-sensor data. Accordingly, employing multiple types of sensors and combining different genres of data outcomes with increasing accuracy on the results.

### 7.2. C-UAV System Recommendation

UAV detection and classification can be an intimidating task because there are many different c-UAV methods available. Nevertheless, a handful of technologies have gradually risen above the rest and been adopted by the majority of researchers and airspace security providers. However, which of the available choices do you pick and why? We outline the pros and cons of each sensor technology and then recommend a potential multi-sensor c-UAV system that can provide the optimal threat’s identification confidence. This recommendation is based on the outcome of the reported work in this literature review, and it focuses on the multi-sensor deep learning fusion of the available data for the UAV detection and classification task. A related deep learning fusion method for c-UAV application that operates with three of the recommended four sensors (radar, electro-optical camera and thermal camera) is reported in [169]. Without loss of generality, this recommendation can be used for other similar surveillance applications that do not target UAVs. For example, the recommendation multi-sensor fusion scheme can adapt to an environmental monitoring application that targets birds that are similar targets to UAVs.

Radar can effectively detect potentially multiple UAVs and track many targets over a long range with constant 360${}^{\circ }$ coverage over a predefined area. Because UAVs are smaller than manned aircraft and tend to fly close to the ground, this makes them very difficult for all but the most specialized radars to detect. Such systems do exist and they usually operate within the *X*-band, but they often present additional issues such as cost (active detection method that requires high electrical power), high-false alarm rate, and potential interference which might require authorizations from local authorities to operate.

Optics allow visual and/or infrared thermal imaging detection and classification of approaching UAVs and potentially identification of UAVs carrying payloads. Optic detection uses cameras to spot intruding UAVs. The cameras can be divided into several types including standard visual security cameras, or thermal cameras which are the most commonly employed for c-UAV systems. Some of the biggest challenges an optic based anti-drone system needs to face are the high false alarm rates and weather-related issues. Cameras have shown consistent issues with false alarms due to the difficulty of differentiating between UAVs and similarly sized airborne objects like birds. Some of these challenges may be mitigated by the complementary use of infrared thermal technology to ferret out UAVs by detecting their heat signatures. However, thermal UAV detection can be adversely affected by weather conditions. High humidity, rain, or dense fog can severely reduce the effectiveness of infrared thermal UAV detection as the infrared radiation is scattered by water particles in the air.

Acoustic UAV detection sensors pick up vibrations made by the propellers and motors of drones and can match them to a database of drone acoustic signatures. Acoustic technology is lightweight, easy to install, and can be used in mountainous or highly urbanized areas where the presence of hillsides or tall buildings might block some other detection methods. It is entirely passive and thus doesn’t interfere with ambient communications and uses little in the way of electric power. However, UAVs are becoming ever more silent as the technology evolves and market pressures demand a quieter device. In addition, acoustic sensors can often detect UAVs, particularly in noisy environments, only at relatively close distances. A single microphone array can detect a drone at less than 150 m so, unless multiple microphone arrays are scattered around the protected area, there is no other way to cover larger distances.

**Figure 10:**
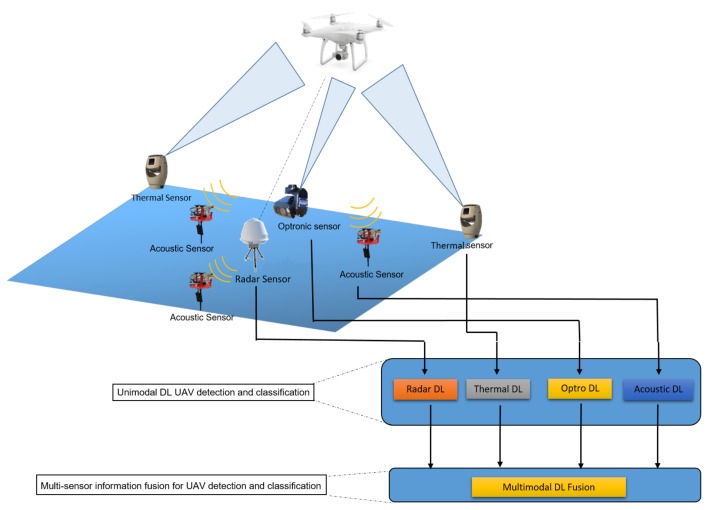
Recommended counter-UAV system.

Each technology has pros and cons but adopting a single sensor for UAV detection and classification will almost certainly not provide the desired situational awareness. Nevertheless, it is possible to find an effective solution, particularly if complementary technologies are mixed (radar and thermal cameras) to assure maximum coverage and secondary technologies (acoustic and optical technologies) to fill in any potential gaps. In Figure 10, we present the recommended c-UAV system that we believe can maintain situational awareness in a robust manner by fusing multiple information from four different sensor types. At the center of the sensor topology, a long range radar is placed. Radar is a reliable way for early detection and it is paired with two panoramic thermal cameras at the edges of the topology to minimize the false alarm rate. The need for more than one thermal cameras is created in order to provide range and azimuth localization of the detected target so as to assist in the fusion of the available information with the radar detections. Due to the sensitivity of the thermal cameras in adverse weather conditions, an optical camera is also placed at the front of the sensor topology to provide an additional means of reducing false alarm when combined with radar and thermal detections. This can be a PTZ (pan tilt zoom) camera that can look at a specific field of view that is given by the radar and thermal detections so as to confirm the presence of a UAV. Finally, in order for the developed c-UAV system to be used in mountainous or highly urbanized areas where the presence of hillsides or tall buildings might block some other detection methods, a number of microphone arrays are also placed scattered around the protected area to cover as much distance as possible and provide another back up solution.

All of the recorded data from each sensor can be utilized by uni-modal deep learning networks developed for UAV detection and classification based on the prevalent methods that were described within the contexts of this literature review. Finally, the unimodal alert signals and the deep learning features that are produced by each uni-modal deep neural network can be fused with a multi-sensor information fusion deep learning network in order to complement each unimodal result and achieve a combined increased confidence in a threat’s identification.

## 8. Conclusions

Research efforts on UAV detection and classification methods based on deep learning using radar, electro-optical, thermal, and acoustic sensors as well as multi-sensor information fusion algorithms have been thoroughly reviewed in the context of this literature review. Research on c-UAV systems is an emerging field and the addition of deep learning may lead to breakthroughs in the years to come. The following section deliberates noteworthy elements from each topic and highlights aspects, which could systematically advance research efforts in the overall field.

Micro Doppler based approaches have shown the most promising detection and classification capabilities. Research on different information sources, such as motion, is not as common, which could encourage further work to be made and provide answers for different operational conditions. Deep learning based object detection algorithms fine-tuned on UAV data are the most common approaches in literature for UAV detection and classification with electro-optical data. Deep learning based object detection and classification architectures have been successfully utilized on thermal imagery for generic targets yet not for UAVs. This could be the motivation for researchers to turn their attention to this novel subject. Recent technological advances in single board computers equipped with GPUs and the low cost of microphones have increased the interest of the researchers in deploying microphones for acoustic classification tasks. In particular, for the task of UAV detection, the deployment of microphone arrays would help in a robust recognition framework, when combined with other sensor modalities.

The application of deep learning on multi-sensor data for UAV detection and classification is considered a rapidly emerging research field. Diverse signals from a variety of sensors can provide significant knowledge aggregation rather than single ones. The scientific publications presented in this work prove the benefits and the necessity of multi-sensor deep learning from a data fusion perspective. The heterogeneity of multi-sensor data leads to challenging constructions of joint representations that exploit inherent relations. Multi-sensor learning employs several techniques for the purpose of efficiently tackling the diversity of data representations. Furthermore, deep learning methods have proved their significance in feature learning and feature representation generation by exhibiting their ability to extract high-level features of different sensors that are semantically correlated. In addition, modern applications of deep learning deal with several multi-sensory data in the form of images, audio, radar signals, etc., which are complicated and require a great deal of effort to learn from them. Consequently, the literature review presented in the field of multi-sensor deep learning sets the basis for a fundamental and promising research field.

## Figures and Tables

**Table 1:** Summary of radar based Unmanned Aerial Vehicle (UAV) detection and classification methods in recent literature

Task	Signal Processing	Classification	Reference
Feature extraction	MDS${}^{1}$ with spectrogram, handcrafted features	-	[19]
Feature extraction	MDS with spectrogram and cepstrogram, handcrafted features	-	[20]
UAV classification	MDS with spectrogram, Eigenpairs extracted from MDS	linear and non linear SVM${}^{2}$, NBC${}^{3}$	[16]
UAV classification, feature extraction	MDS with spectrogram, cepstrogram and CVD${}^{4}$, SVD${}^{5}$ on MDS	SVM	[21,22]
UAV classification, feature extraction	MDS with 2D regularized complex-log-Fourier transform	Subspace reliability analysis	[23]
UAV classification, feature extraction	MDS with EMD${}^{6}$, features from EMD	SVM	[24]
UAV classification, feature extraction	MDS with EMD, entropy from EMD features	SVM	[25]
UAV classification, localization	MDS with EMD, PCA${}^{7}$ on MDS	Nearest Neighbor, NBC, random forest, SVM	[26]
UAV classification	MDS with spectrogram, handcrafted features	NBC, DAC${}^{8}$	[27]
UAV detection, tracking	MDS with spectrogram, CFAR${}^{9}$ for detection, Kalman for tracking	-	[28]
UAV classification, feature extraction	MDS with spectrogram, PCA on MDS	SVM	[29]
UAV trajectory classification	Features from moving direction, velocity, and position of the target	Probabilistic motion estimation model	[30]
UAV trajectory and type classification, feature extraction	Features from motion, velocity, signature	SVM	[31]
UAV classification, feature extraction	Radar polarimetric features	Nearest Neighbor	[32]
UAV classification	MDS with spectrogram and CVD	CNN${}^{10}$	[33]
UAV classification	SCF${}^{11}$ reference banks	DBN${}^{12}$	[34]
Target detection	Doppler processing	CNN	[35]
UAV classification	Direct learning on Range Profile matrix	CNN	[36]
UAV classification	Direct learning on IQ${}^{13}$ signal	MLP${}^{14}$	[37]
UAV classification	Point cloud from radar signal	MLP	[38]
UAV trajectory classification, feature extraction	Features from motion, velocity, RCS${}^{15}$	MLP	[39]

MDS${}^{1}$: Micro Doppler Signature, SVM${}^{2}$: Support Vector Machine, NBC${}^{3}$: Naive Bayes Classifier, CVD${}^{4}$: Cadence Velocity Diagram, SVD${}^{5}$: Singular Value Decomposition, EMD${}^{6}$: Empirical Mode Decomposition, PCA${}^{7}$: Principal Component Analysis, DAC${}^{8}$: Discriminant Analysis Classifier, CFAR${}^{9}$: Constant False Alarm Rate, CNN${}^{10}$: Convolutional Neural Network, SCF${}^{11}$: Spectral Correlation Function, DBN${}^{12}$: Deep Belief Network, IQ${}^{13}$: In-phase and Quadrature, MLP${}^{14}$: Multi Layer Perceptron, RCS${}^{15}$: Radar Cross Section.

**Table 2:** Results of recent radar based Unmanned Aerial Vehicle (UAV) classification methods

Classification Task (Num. of Classes)	Classification Method	Accuracy (%)	Reference
UAV type vs. birds (11)	Eigenpairs of MDS${}^{1}$ + non linear SVM${}^{2}$	82 ${}^{*}$	[16]
UAV type vs. birds (11)	MDS with EMD${}^{3}$ + SVM	89.54 ${}^{*}$	[24]
UAV type vs. birds (11)	MDS with EMD, entropy from EMD + SVM	92.61 ${}^{*}$	[25]
UAV vs. birds (2)	SVD${}^{4}$ on MDS + SVM	100	[22]
UAV type (2)	SVD on MDS + SVM	96.2	[22]
UAV vs. birds (2)	2D regularized complex log-Fourier transform + Subspace reliability analysis	96.73	[23]
UAV type + localization (66) ${}^{**}$	PCA${}^{5}$ on MDS + random forest	91.2	[26]
loaded vs. unloaded UAV (3)	MDS handcrafted features + DAC${}^{6}$	100	[27]
UAV type (3)	PCA on MDS + SVM	97.6	[29]
UAV type vs. birds (4)	Radar polarimetric features + Nearest Neighbor	99.2	[32]
UAV vs. birds (2)	Range Profile Matrix + CNN${}^{7}$	95	[36]
UAV type (6)	MDS and CVD${}^{8}$ images + CNN	99.59	[33]
UAV type vs. birds (3)	SCF${}^{9}$ reference banks + DBN${}^{10}$	90	[34]
UAV type (2)	Learning on IQ${}^{11}$ signal + MLP${}^{12}$	100	[37]
UAV type (3)	Point cloud features + MLP	99.3	[38]
UAV vs. birds (2)	Motion, velocity and RCS${}^{13}$ features + MLP	99	[39]
UAV type vs. birds (3)	Motion, velocity and signature features + SVM	98	[31]

MDS${}^{1}$: Micro Doppler Signature, SVM${}^{2}$: Support Vector Machine, EMD${}^{3}$: Empirical Mode Decomposition, SVD${}^{4}$: Singular Value Decomposition, PCA${}^{5}$: Principal Component Analysis, DAC${}^{6}$: Discriminant Analysis Classifier, CNN${}^{7}$: Convolutional Neural Network, CVD${}^{8}$: Cadence Velocity Diagram, SCF${}^{11}$: Spectral Correlation Function, DBN${}^{10}$: Deep Belief Network, IQ${}^{11}$: In-phase and Quadrature, MLP${}^{12}$: Multi Layer Perceptron, RCS${}^{13}$: Radar Cross Section. ${}^{*}$ These numbers stand for comparable dwell time on the order of <0.25 s; ${}^{**}$ Two UAV types, with 35 and 31 locations under test respectively.
